# Left Atrial Appendage Thrombus in Patients with Nonvalvular Atrial Fibrillation before Catheter Ablation and Cardioversion: Risk Factors beyond the CHA2DS2-VASc Score

**DOI:** 10.3390/jcdd9020046

**Published:** 2022-01-30

**Authors:** Yangwei Cai, Qingsong Xiong, Shaojie Chen, Xi Jiang, Jia Liao, Weijie Chen, Lili Zou, Lei Su, Yefeng Zhu, Yuehui Yin, Zhiyu Ling

**Affiliations:** 1 Department of Cardiology, Second Affiliated Hospital of Chongqing Medical University, Chongqing 400010, China; yangweicai@stu.cqmu.edu.cn (Y.C.); qingsongdr@163.com (Q.X.); drsjchen@126.com (S.C.); hsikwong@163.com (X.J.); JLiao@stu.cqmu.edu.cn (J.L.); cqmucwj@hospital.cqmu.edu.cn (W.C.); LilyZou@cqmu.edu.cn (L.Z.); nicolsue@163.com (L.S.); zhuyefeng0575@163.com (Y.Z.); yinyh63@163.com (Y.Y.); 2 Cardioangiologisches Centrum Bethanien (CCB), Kardiologie, Markus Krankenhaus, 60308 Frankfurt am Main, Germany

**Keywords:** nonvalvular atrial fibrillation, left atrial appendage thrombus, risk factors, CHA2DS2-VASc score

## Abstract

Left atrial appendage thrombus (LAAT) is a surrogate of thromboembolic events in patients with nonvalvular atrial fibrillation (NVAF). We aimed to investigate the risk factors for LAAT formation before catheter ablation and cardioversion beside the CHA2DS2-VASc score. In this case-control study, patients with NVAF who underwent transesophageal echocardiography (TEE) were included. Demographic data, laboratory results, and echocardiographic measurements were retrospectively collected. Logistic regression analysis was performed to determine risk factors predicting LAAT. Of the 543 included patients, LAAT was identified in 50 patients (9.2%). Multivariable logistic regression analysis for the entire cohort showed that NT-proBNP (per 500 ng/L increase, OR (95% CI): 1.09 (1.00–1.19), *p* = 0.038) and LDL-C (per 1 mmol/L increase, OR (95% CI): 1.70 (1.05–2.77), *p* = 0.032) were independently correlated with the presence of LAAT after the adjustment for CHA2DS2-VASc score and anticoagulant therapy. The subgroup analysis of patients without anticoagulant therapy also yielded similar results. Regarding patients with CHA2DS2-VASc scores ≤ 1, a higher level of LDL-C (per 1 mmol/L increase, OR (95% CI): 6.31 (2.38–16.74), *p* < 0.001) independently correlated with the presence of LAAT. The present study suggests that beyond CHA2DS2-VASc score, raised NT-proBNP and LDL-C are additional predictors for LAAT in NVAF patients.

## 1. Introduction

Atrial fibrillation (AF) is the most common cardiac arrhythmia worldwide, with a currently estimated prevalence between 2–4% in adults, which is expected to continue rising in the near future [1]. The presence of AF is intimately correlated to cardiovascular hospitalizations and all-cause mortality, even nullifying the prognostic power of cardiac calcification in patients with multiple cardiovascular risk factors [2]. Stroke is the most clinically significant complication observed in patients with AF, with a risk 4–5 times higher than patients without. Stroke can be fatal (up to 20%) or severely disabling (about 60%) [3]. It has been well recognized that the left atrial appendage thrombus (LAAT) formation is the primary cause of cardiogenic stroke in patients with nonvalvular atrial fibrillation (NVAF) [4].

CHA2DS2-VASc score is currently the most commonly used scoring system for the risk stratification of stroke in patients with NVAF [1,5], which exhibits comparable predictive value even in patients with sinus rhythm [6]. However, the CHA2DS2VASc score is based solely on demographic variables and shows modest prognostic performance [7,8]. It remains controversial whether anticoagulant therapy should be administrated in patients with CHA2DS2-VASc ≤ 1 (CHA2DS2-VASc ≤ 2 in women) in consideration of balancing the risk and efficacy in this selected patient group [9]. Evidence has also shown a certain incidence of thromboembolism, even in patients receiving anticoagulant therapy [8,10,11].

Prior works have well established that biomarkers including NT-proBNP [12,13], cTNI [12,14], eGFR [15,16], D-dimer [12,17], and LDL-C [18] have a positive predictive value for stroke and systemic embolism in patients with NVAF. Additionally, echocardiographic parameters on cardiac functional and structural status, for instance, left ventricular ejection fraction(LVEF, %) [19], left atrial appendage flow velocity(<20 cm/s) [20], and left atrial enlargement [21] are also predictors for stroke risk in patients with NVAF. The derived ABC-stroke score (age, troponin, NT-proBNP and clinical history of stroke/TIA) [7,8] and R2CHADS2 score (supplementing the CHADS2 score with an additional 2 points for CrCl < 60 mL/min) [15] outperformed CHA2DS2-VASc score and demonstrated the value of risk factors beyond CHA2DS2-VASc score for refining stroke risk stratification in patients with NVAF. 

As left atrial appendage thrombus (LAAT) is considered as a surrogate of thromboembolic events, in this study we aimed to investigate potential risk factors beyond CHA2DS2-VASc score in predicting LAAT in patients with NVAF undergoing transesophageal echocardiography (TEE) before catheter ablation and cardioversion.

## 2. Methods

### 2.1. Study Population

Consecutive patients with documented NVAF who were scheduled for cardioversion (catheter ablation and electrical cardioversion) therapy and underwent transesophageal echocardiography (TEE) were retrospectively collected at the Second Affiliated Hospital of Chongqing Medical University. Patients with LAAT from May 2015 until August 2020 and those without LAAT from September 2018 until August 2020 were retrospectively collected. All clinical, laboratory and echocardiographic data were obtained retrospectively from medical records. The study was approved by the Ethics Committee of the Chongqing Medical University’s Second Affiliated Hospital. The committee waived the need for informed consent because of the observational nature of the study.

### 2.2. Clinical and Laboratory Biomarkers Data

Baseline characteristics involving age, gender, body mass index (BMI), AF type, AF duration, presence and type of oral anticoagulant (OAC), comorbidities including congestive heart failure, hypertension, diabetes mellitus, stroke/TIA/thromboembolism, vascular disease, renal impairment and anticoagulant duration before TEE were retrieved from medical records. The type of AF was classified into paroxysmal AF and non-paroxysmal AF (persistent AF or long-standing persistent AF). AF duration was defined as the period from the first time of documented AF to the day of admission. All CHA2DS2-VASc scores after admission were recalculated by two investigators (Y.C. and Q.X.) blinded to the LAAT status according to the scoring metric newly updated in the 2020 ESC guidelines [1], and conflicts were resolved by discussion. The presence of anticoagulant therapy was defined as continuously receiving oral anticoagulant drugs for at least 3 weeks before the TEE examination. All blood samples for testing biomarkers involving N-terminal pro-B-type natriuretic peptide (NT-proBNP), cardiac troponin I (cTNI), creatine kinase isoenzyme (CK-MB), estimated glomerular filtration rate (eGFR), uric acid, triglycerides (TG), total cholesterol (TC), and low-density lipoprotein cholesterol (LDL-C) were collected after admission and before the TEE examination. The eGFR was calculated using the Modification of Diet in Renal Disease (MDRD) Study equation [22], renal impairment was defined as eGFR < 60 mL/min/1.73 m^2^ according to the recommendation of chronic kidney disease clinical practice guideline developed by Kidney Disease: Improving Global Outcomes (KDIGO) [23].

### 2.3. Echocardiographic Data

After admission, TEE was performed by two well-experienced attending physicians to detect thrombus formation in LAA/LA before electrical cardioversion or catheter ablation, even in patients treated with anticoagulation for more than 3 weeks. LAA/LA thrombus was defined as a well-circumscribed echodense intracavitary mass distinct from the underlying endocardium and not related to pectinate muscles [24]. Suspected thrombus was observed from different angles (0°, 45°, 90°, 135°) and evaluated by two qualified attending physicians. Discrepancies were resolved by consensus discussion or by an expertized physician if needed. Left atrial diameter (LAD, determined as the posterior aortic wall to the posterior left atrial wall at the end-ventricular systole) and left ventricular ejection fraction (LVEF) were routinely measured from M-mode or 2D view in the parasternal long-axis projection under transthoracic echocardiography before TEE [25].

### 2.4. Statistical Analysis

Normally distributed continuous variables were expressed as mean ± standard deviations, while non-normally distributed continuous variables were expressed as median (interquartile ranges), and categorical variables as numbers and percentages (%). Normal distribution was verified by the Shapiro–Wilk test. The continuous variables were compared by Student *t*-test for normally distributed data, and the Mann–Whitney U-test was used for non-normally distributed data. For comparison of categorical variables, the chi-square test or Fisher’s exact test was utilized appropriately. For those variables with *p* < 0.10 (to avoid omission of potential risk factors) at univariable analysis and clinically relevant variables (CHA2DS2-VASc score and use of OAC), multivariable logistic regressions were performed to determine the independent predictors for LAAT in the entire cohort, CHA2DS2-VASc score ≤ 1, and patients without OAC subgroups, respectively. Certain variables were included in the CHA2DS2VASc score, such as gender, hypertension, heart failure, LVEF, and vascular disease, although *p* < 0.1 were not repeatedly entered into the multivariable analysis to avoid the potential interactions with CHA2DS2-VASc score. The Box–Tidwell Test was utilized to ascertain the linear relationship between any continuous independent variables and the logit transformed dependent variable. The variance inflation factor was used to determine the collinearity of variables and values of >5 was considered suggestive of collinearity. ROC curves were drawn for the entire cohort and subgroups separately, and the AUCs were calculated to determine the incremental value of the identified risk factors in predicting LAAT comparing with the original CHA2DS2-VASc score. All significance tests were two-tailed and *p* values < 0.05 were considered statistically significant. All statistical analyses were performed using SPSS 26.0 (SPSS 26.0J, Chicago, IL, USA).

## 3. Results

### 3.1. Baseline Characteristics

A total of 543 patients (male = 284, 57.6%) who underwent TEE before catheter ablation and cardioversion were consecutively included. LAA thrombi were found in 50 patients (one was detected with concomitant LAA and LA thrombus). Among the individuals without LAAT (*n* = 493), 75.9% (*n =* 374) underwent catheter ablation, and 24.1% (*n* = 119) received electrical cardioversion. Baseline characteristics of the two groups (non-LAAT group, *n* = 493; and LAAT group, *n* = 50) are presented in Table 1. There were no significant differences in age, BMI, AF classification, length of AF history, smoke history, alcohol history, renal repairment, and CHA2DS2-VASc scores between the two groups. Significantly, the LAAT group had higher rates of hypertension (*p* = 0.042) and heart failure (*p* < 0.001), and a lower rate of anticoagulant therapy (*p* = 0.009). There was no difference in the anticoagulant duration before TEE for both groups (*p* = 0.944). In addition, there were more patients in AF rhythm at the time of TEE in LAAT group (*p* = 0.001).

There were no significant differences in the baseline characteristics between the non-LAAT and LAAT subgroups with a CHA2DS2-VASc score ≤ 1 (non-LAAT, *n* = 228; LAAT, *n* = 20). Similar to the results of the entire cohort, LAAT patients more often had heart failure (*p* < 0.001) in the subgroup analysis of patients without OAC (non-LAAT, *n* = 331; LAAT, *n* = 44). There was a significantly higher incidence of AF rhythm at TEE in the LAAT group in both subgroup analyses (*p* = 0.026 and *p* < 0.001, respectively), as shown in Table 2.

### 3.2. Laboratory Serum Biomarkers and Echocardiographic Data

Table 3 shows the comparisons of serum biomarkers and echocardiographic data between the Non-LAAT and the LAAT group. For the entire cohort, the LAAT group had significantly higher levels of NT-proBNP (1352.90 (508.50–2828.00) vs. 649.90 (231.00–1310.70) ng/L, *p* < 0.001), cTNI (10.00 (6.00–22.00) vs. 8.00 (1.00–10.00) ng/L, *p* < 0.001), LDL-C (2.24 ± 0.75 vs. 2.04 ± 0.63 mmol/L, *p* = 0.038) and a lower level of eGFR (79.89 ± 21.52 vs. 87.97 ± 22.81 mL/min/1.73 m^2^, *p* = 0.017) when compared with the non-LAAT group. Regarding the echocardiography data, the LAAT group had larger left atrial diameters (LAD, 42.14 ± 6.67 vs. 38.94 ± 5.38 mm, *p* < 0.001) and lower LVEF (59.92 ± 11.91% vs. 65.29 ± 9.47%, *p* < 0.001). The same trend was also observed in the subgroup analysis of patients without OAC.

In the subgroup of CHA2DS2-VASc score ≤ 1, the LAAT group had a higher level of cTNI (7.00 (3.00–21.00) vs. 1.00 (1.00–10.00) ng/L, *p* = 0.027), uric acid (414.50 (335.50–483.50) vs. 346.65 (274.25–401.25) mmol/L, *p* = 0.005), LDL-C (2.78 ± 0.62 vs. 2.18 ± 0.54 mmol/L, *p* < 0.001), and lower LVEF (59.50 ± 10.64 vs. 65.02 ± 8.82%, *p* = 0.009), as compared with the non-LAAT group.

### 3.3. Multivariable Logistic Regression

**Figure 1:**
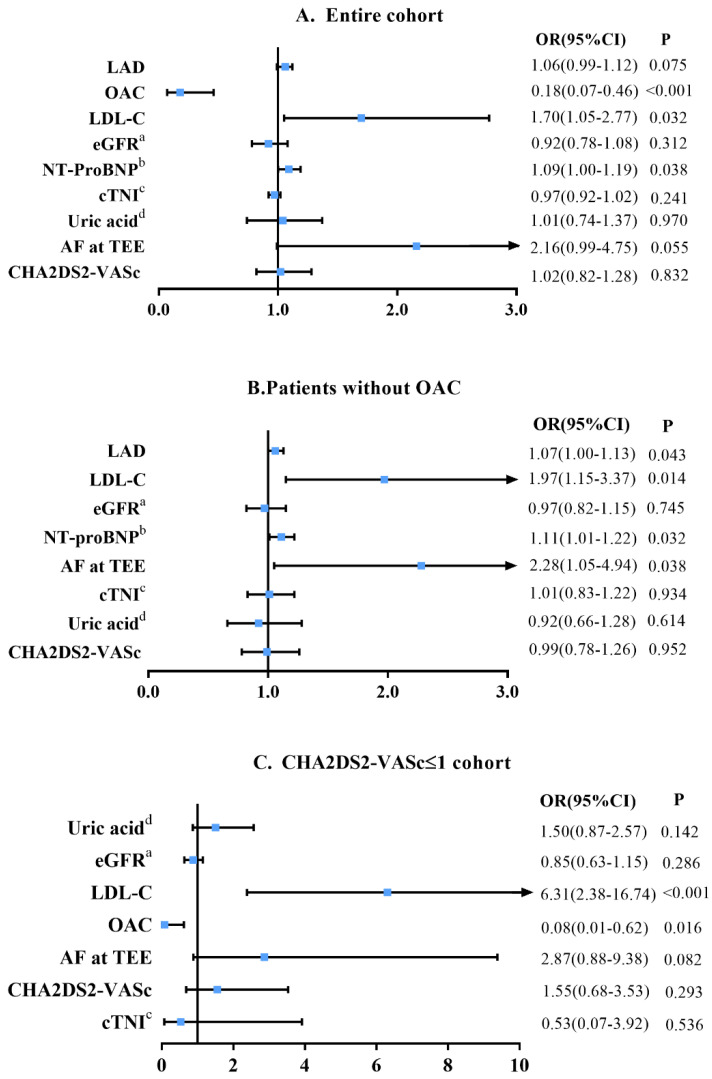
Forest plot for odds ratio in multivariable logistic regressions. (**A**) Entire cohort; (**B**) patients without OAC; (**C**) CHA2DS2-VASc ≤ 1 cohort. a: eGFR (per 10 mL/min/1.73 m^2^ increase); b: NT-proBNP (per 500 ng/L increase); c: cTNI (per 100 ng/L increase); d: uric acid (per 100 mmol/L increase). Abbreviations: OAC = oral anticoagulant; eGFR = estimated glomerular filtration rate; LAD = left atrial diameter; NT-proBNP = N-terminal pro-B-type natriuretic peptide; LDL-C = low-density lipoprotein cholesterol; cTNI = cardiac troponin I.

We further performed the multivariable logistic regression for all variables with *p* < 0.10 and the CHA2DS2-VASc score to identify potential risks factors of LAAT. Variables already included in the CHA2DS2VASc score, such as sex, were not repeatedly entered into the multivariable model. Variance inflation factors for all variables were less than 5, indicating no collinearity. Figure 1A reveals that the levels of NT-proBNP (per 500 ng/L increase, OR (95% CI): 1.09 (1.00–1.19), *p* = 0.038) and LDL-C (per 1 mmol/L increase, OR (95% CI): 1.70 (1.05–2.77), *p* = 0.032) remained independently associated with increased risk of LAAT after the adjustment of the CHA2DS2-VASc score, and the use of OAC was a protective factor for LAAT (OR (95% CI): 0.18 (0.07–0.46), *p* < 0.001). We further conducted the multivariable analysis in subgroups of patients without OAC (Figure 1B). Similar results with NT-proBNP and LDL-C as risk factors were also observed. Additionally, LAD (OR (95% CI): 1.07 (1.00–1.13), *p* = 0.043) and AF rhythm at TEE (OR (95% CI): 2.28 (1.05–4.94), *p* = 0.038) also showed an association with LAAT in patients without anticoagulant therapy.

With regard to the subgroups of CHA2DS2-VASc score ≤ 1 (female excluded), cTNI, LDL-C, uric acid, eGFR, rhythm at TEE, CHA2DS2-VASc score and use of OAC were finally enrolled into the multivariable analysis model. Results showed that LDL-C (per 1 mmol/L increase, OR (95% CI): 6.31 (2.38–16.74), *p* < 0.001) was independent risk factors of LAAT in this subgroup (Figure 1C). Notably, CHA2DS2-VASc score showed no significant correlation with the presence of LAAT in all multivariable logistic regression models.

### 3.4. ROC Analyses

**Figure 2:**
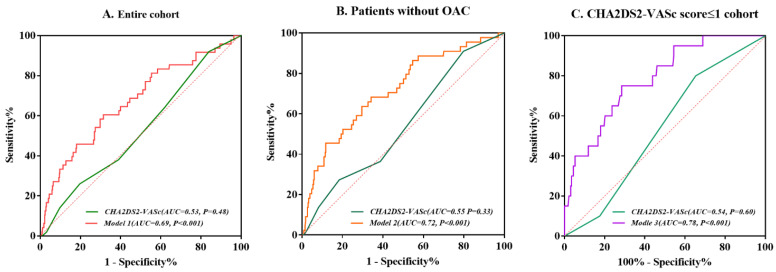
Receiver operating characteristic (ROC) curves for the prediction of LAAT in the entire cohort (**A**)**,** patients without OAC (**B**) and CHA2DS2-VASc score ≤ 1 cohort (**C**). Model 1: CHA2D2S2-VASc score incorporated with NT-proBNP and LDL-C for entire cohort; Model 2: CHA2D2S2-VASc score incorporated with NT-proBNP and LDL-C for patients without OAC; Model 3: CHA2D2S2-VASc score incorporated with LDL-C for CHA2DS2-VASc score ≤ 1 cohort.

The receiver operator characteristic (ROC) curves demonstrated that NT-proBNP and LDL-C incorporated into the CHA2DS2-VASc score (AUC (95% CI): 0.69 (0.60–0.77) *p* < 0.001) improved the predictive ability of LAAT, compared with the original CHA2DS2-VASc score alone (AUC (95% CI): 0.53 (0.45–0.61), *p* = 0.48) in the entire cohort (Figure 2A). The incremental predictive value of NT-proBNP and LDL-C was also reflected in the subgroup analysis of patients without OAC with an AUC of 0.72 (95% CI: 0.63–0.80, *p* < 0.001) (Figure 2B). Similarly, the ROC analysis for the CHA2DS2-VASc score ≤ 1 cohort revealed that CHA2DS2-VASc score plus LDL-C was superior to the score system alone, in predicting LAAT with a higher AUC of 0.78 (95% CI: 0.69–0.88, *p* < 0.001), compared to 0.54 (95% CI: 0.42–0.65, *p* = 0.60) (Figure 2C).

### 3.5. Anticoagulant Therapy before TEE and Outcomes of Patients with LAAT

Before TEE, anticoagulant therapy was performed in 145 patients without LAAT for 8 (4–31) weeks: warfarin (1.25–3.75 mg qd for 3 patients), dabigatran (110 mg bid for 71 patients and 150 mg bid for 6 patients), and rivaroxaban (15 mg qd for 59 patients and 20 mg qd for 6 patients). For patients with LAAT, 6 patients had also received anticoagulation for 12 (7–19) weeks before TEE: warfarin (2.5 mg qd for 2 patients), dabigatran (110 mg bid for 1 patient and 150 mg bid for 1 patient), and rivaroxaban (15 mg qd for 2 patients).

All 50 patients received oral anticoagulant therapy after diagnosis of LAAT, including 21 patients with rivaroxaban, 12 patients with warfarin, and 17 patients with dabigatran. Moreover, 22 patients reviewed TEE after anticoagulation treatment for a median of 94.5 days (interquartile range: 76–130), and the results showed that thrombi resolved in 12 patients. Of the remaining patients with still existing LAAT and those without reexamination of TEE, thromboembolic events occurred in 2 patients, and 2 patients died within 16.3 months (interquartile range: 8.9–24.3) (Table 4).

## 4. Discussion

The present study enrolled consecutive patients with NVAF undergoing TEE before catheter ablation and electrical cardioversion from a single center to identify factors that affect LAA thrombogenesis, especially in patients with low CHA2DS2-VASc scores. After adjusting for CHA2DS2-VASc score and oral anticoagulation treatment factors, we concluded: (1) elevated NT-proBNP and LDL-C were independent risk factors for LAAT in patients with NVAF beyond the CHA2DS2-VASc score and anticoagulation therapy; (2) in particular, LDL-C showed excellent independent prediction ability for LAAT formation in patients with lower CHA2DS2-VASc scores.

Consistent with the findings in previously mentioned studies focusing on the endpoint of stroke or systemic embolism [12,13,26], in this study we demonstrated the similar predictive value of NT-proBNP for LAAT detected by TEE in patients with NVAF. The concept of fibrotic atrial cardiomyopathy provides a potential mechanistic explanation for the links between NT-proBNP and LAAT [27]. The serum level of NT-proBNP as a marker of myocyte stress may also reflect the degree of atrial myopathy, which is recognized as an atrial substrate for thrombosis, even independently of atrial rhythm and heart failure.

As an important factor in the occurrence and progression of atherosclerosis, LDL-C has been widely validated as playing a vital role in the prediction of stroke for all populations [28]. Prior works have reported LDL-C as an independent risk factor for ischemic stroke among patients with AF [29,30]. In a case-controlled study, Qi Z et al. [30] confirmed the dose–response pattern association between LDL-C and ischemic stroke in patients with AF, and showed that the addition of LDL-C information to CHA2DS2-VASc score could provide an incremental predictive value for the risk of ischemic stroke. Furthermore, a later study showed that lowering LDL-C could be particularly beneficial among AF patients with a low CHA2DS2VASc score (less than 2 in males and 3 in females) [31]. Consistent with this finding, our present study found that raised LDL-C was also an independent predictor of LAAT formation among NVAF patients with a low CHA2DS2-VASc score. To the best of our knowledge, this is the first direct evidence to demonstrate that LDL-C is implicated in thrombogenesis in AF, beyond its traditional role in atherosclerosis, which may also cause stroke. The underlying mechanism may be that LDL-C interacts with hemostatic pathways to promote excess platelet activation and thrombin generation, as well as inhibiting fibrinolysis [18]. For the actuality that LDL-C is a risk predictor for LAAT, whether lipid-lowering therapy can prevent patients with NVAF from LAAT remains unclear, and further exploration is required.

In addition, unlike previous studies [32,33,34], uric acid and BMI have not been shown to influence LAAT formation independently in the current study, although previous studies have fully demonstrated the independent predictive value of the impaired renal function for thromboembolic events in patients with atrial fibrillation [15,16]. Our study did not find the association of eGFR with LAAT after adjustment for CHA2DS2-VASc score and use of OAC. These discrepancies are likely because we enrolled relatively healthier patients who were scheduled for electrical cardioversion and catheter ablation therapy.

It is worth noting that up to 40% of the patients with LAAT formation had a non-sex-related CHA2DS2-VASc score ≤ 1 in the present study, and there was no significant correlation between CHA2DS2-VASc score and the presence of LAAT in multivariable logistic regression analyses. This may be attributed to the fact that the CHA2DS2-VASc risk score is solely based on clinical variables with a modest prognostic performance [35]; patients with LAAT with lower CHA2DS2-VASc scores may be affected by potential risk factors beyond CHA2DS2-VASc score. On the other hand, the low anticoagulation rate was one crucial explanatory factor for the high incidence of LAAT in patients with low CHA2DS2-VASc scores. Moreover, in this study, six (12%) patients with anticoagulation for more than 3 weeks were still detected with LAA thrombus. This is consistent with a recent meta-analysis that revealed a nonnegligible prevalence of LA thrombus, even in well-anticoagulated patients with AF, in which a mean-weighted prevalence of 2.73% (95% CI: 1.95–3.80%) among 14,653 patients was reported [36]. This indicates the necessity of performing TEE before cardioversion or catheter ablation, even in patients with low-intermediate risk for stroke before TEE, or those who have received anticoagulant therapy for more than 3 weeks.

Oral anticoagulants are recommended for an elevated CHA2DS2-VASc score ≥ 2 in men or ≥3 in women. In contrast, it is reasonable to omit anticoagulant therapy for patients with scores of 0 in men, or 1 in women [1,37]. However, it remains controversial whether anticoagulant therapy should be conducted in patients with a CHA2DS2-VASc score of 1 (CHA2DS2-VASc of 2 in women), because of the major challenge in balancing the risk of stroke and bleeding in this population [9]. Adjusted annual stroke risk in NVAF patients presenting with a CHA2DS2-VASc score of 1 had been reported as 0.6–1.3% in the trial cohort from which the CHA2DS2-VASc score originated [38]. Hence, there is a controversy in the anticoagulant therapy decision for this population based on the common consensus that a thromboembolic event rate of 1% per year is needed to justify the initiation of OAC in AF [39]. The actual stroke risk for individuals in this cohort may differ; thus, a more accurate assessment of stroke risk is needed. Our study found that NT-proBNP and LDL-C were independent risk factors of LAAT formation for NVAF individuals and LDL-C remained significantly associated with the presence of LAAT in non-sex-related CHA2DS2-VASc ≤ 1 and patients without OAC subgroup analyses. The ROC analyses for the entire cohort and subgroups also presented significantly incremental predictive valued of NT-proBNP and LDL-C for LAAT, compared with CHA2DS2-VASc score alone. This suggests that NT-proBNP and LDL-C could be potentially used to further refine stroke risk stratification in NVAF patients, especially those judged as suffering low–intermediate stroke risk by CHA2DS2-VASc score. Individualized anticoagulation decisions will be made to enable patients to benefit further from anticoagulant therapy.

Several limitations of this analysis should be acknowledged. This was a single-center retrospective observational study with a medium sample size, the causal relationship could not be clearly defined and the relatively limited sample size of patients with LAAT might reduce the statistical power. Secondly, body size index (BMI), D-dimer, C-reactive protein, left atrial volume, left atrial appendage emptying velocity, left atrial appendage morphology, and other factors, were not collected due to the lack of clinical data. Thirdly, due to the lack of data on statin use, the effect of lipid-lowering therapy on left atrial appendage thrombosis remains uncertain. In addition, the patients with LAA spontaneous echocontrast (SEC) detected in TEE were not separately included into the comparative analysis. Moreover, all patients we included were scheduled for cardioversion therapy and might have been relatively healthier. Therefore, the results of the present study may not be directly applicable to the general atrial fibrillation population. Finally, all the patients were from the southwest of China; studies have shown people with atrial fibrillation in Asia have a higher risk of embolism, so the result of this study may not apply to other populations, such as Caucasians or African Americans.

## 5. Conclusions

This study demonstrated that raised NT-proBNP and LDL-C were significantly associated with the presence of LAAT in NVAF patients before catheter ablation and electrical cardioversion after the adjustment for CHA2DS2-VASc score and anticoagulation therapy. Specifically, higher levels of LDL-C were independent risk factors for LAAT in patients with non-sex-related CHA2DS2-VASc score ≤ 1. Further study is required to explore the role of these factors in the LAA thrombogenesis of patients with NVAF. In addition, it is reasonable to perform transesophageal echocardiography before catheter ablation or cardioversion therapy in patients with non-sex-related CHA2DS2-VASc score ≤ 1, or those receiving anticoagulant therapy.

## Figures and Tables

**Table 1:** Baseline characteristics of both groups for entire cohort.

Characteristics	Non-LAAT (*n* = 493)	LAAT (*n* = 50)	*p*-Value
Sex (male%)	284 (57.6%)	35 (70.0%)	0.090
Age (years)	65 (55–70)	64 (53–70)	0.675
Paroxysmal (%)	293 (59.4%)	28 (56%)	0.638
Non-Paroxysmal (%)	200 (40.6%)	22 (44.0%)	0.638
Length of AF history (month)	24 (2–60)	36 (6–72)	0.281
Body mass index (kg/m^2^)	24.39 ± 3.17	24.73 ± 3.60	0.482
Smoke history (%)	161 (32.7%)	21 (42.0%)	0.182
Alcohol history (%)	102 (20.7%)	13 (26.0%)	0.381
Hypertension (%)	251 (50.9%)	33 (66.0%)	0.042
Diabetes mellitus (%)	88 (17.8%)	8 (16.0%)	0.744
Vascular disease (%)	75 (15.2%)	9 (18.0%)	0.604
Coronary artery disease (%)	47 (9.5%)	8 (16.0%)	0.149
Thromboembolic events (%)	45 (9.1%)	5 (10.0%)	0.839
Heart failure (%)	42 (8.5%)	13 (26.0%)	<0.001
Renal impairment (%)	45 (9.1%)	9 (18.0%)	0.077
CHA2DS2-VASc ≥ 2 (female excluded)	265 (53.8%)	30 (60.0%)	0.398
CHA2DS2-VASc score	2.18 ± 1.62	2.36 ± 1.59	0.474
Anticoagulant therapy (%)	162 (32.86%)	6 (12.0%)	0.009
Warfarin (%)	20 (4.06%)	2 (4.0%)	0.069
Dabigatran (%)	77 (15.6%)	2 (4.0%)	0.026
Rivaroxaban (%)	65 (13.2%)	2 (4.0%)	0.060
NOAC (%)	142 (28.8%)	4 (8.0%)	0.002
Anticoagulant duration before TEE (weeks)	8 (4–31)	12 (7–19)	0.944
Sinus rhythm at TEE	237 (48.1%)	12 (24.0%)	0.001
AF rhythm at TEE	256 (51.9%)	38 (76.0%)	0.001

Abbreviations: AF, atrial fibrillation; NOAC, novel oral anticoagulant.

**Table 2:** Baseline characteristics of subgroups with CHA2DS2-VASc score ≤1 (female excluded) and patients without OAC.

Characteristics	Patients with CHA2DS2-VASc Score ≤ 1			Patients without OAC		
	Non-LAAT (*n* = 228)	LAAT (*n* = 20)	*p*-Value	Non-LAAT (*n* = 331)	LAAT (*n* = 44)	*p*-Value
Sex (male%)	146 (64.0%)	16 (80.0%)	0.220	192 (58.01%)	30 (68.18%)	0.197
Age (years)	56 (50–63)	54 (50–63)	0.548	64 (54–70)	63 (53–69)	0.856
Paroxysmal (%)	138 (60.5%)	12 (60.0%)	0.963	214 (64.65%)	27 (61.36%)	0.669
Non-Paroxysmal (%)	90 (39.5%)	8 (40.0%)	0.963	117 (35.35%)	17 (38.64%)	0.669
Length of AF History (Month)	16 (3–48)	48 (5–81)	0.102	24 (3–48)	36 (6–72)	0.188
Body Mass Index (kg/m^2^)	24.44 ± 3.05	24.34 ± 4.01	0.885	24.51 ± 3.16	25.12 ± 3.32	0.233
Smoke History (%)	85 (37.3%)	9 (45.0%)	0.495	106 (32.02%)	19 (43.18%)	0.140
Alcohol History (%)	60 (26.3%)	7 (35.0%)	0.402	68 (20.54%)	12 (27.27%)	0.306
Hypertension (%)	44 (19.3%)	6 (30.0%)	0.253	160 (48.34%)	28 (63.64%)	0.057
Diabetes Mellitus (%)	8 (3.5%)	2 (10.0%)	0.188	58 (17.52%)	7 (15.91%)	0.791
Vascular Disease (%)	4 (1.8%)	2 (10.0%)	0.076	46 (13.90%)	7 (15.91%)	0.719
Heart Failure (%)	4 (1.8%)	1 (5.0%)	0.346	25 (7.55%)	13 (29.55%)	<0.001
Renal Impairment	9 (3.9%)	2 (10.0%)	0.219	30 (9.1%)	7 (15.9%)	0.153
Stroke/TIA	-	-	-	25 (7.55%)	4 (9.09%)	0.720
Anticoagulant Therapy (%)	72 (31.6%)	3 (15.0%)	0.137	-	-	-
Warfarin (%)	13 (5.7%)	2 (10.0%)	0.345	-	-	-
Dabigatran (%)	34 (15.4%)	1 (5.0%)	0.324	-	-	-
Rivaroxaban (%)	24 (10.5%)	0 (0%)	0.233	-	-	-
NOAC (%)	59 (25.9%)	1 (5.0%)	0.052	-	-	-
CHA2DS2-VASc Score	0.83 ± 0.70	0.90 ± 0.55	0.595	2.1 ± 1.6	2.3 ± 1.6	0.318
Sinus Rhythm at TEE	116 (50.9%)	5 (25.0%)	0.026	141 (42.60%)	32 (72.73%)	<0.001
AF Rhythm at TEE	112 (49.1%)	15 (75.0%)	0.026	190 (57.40%)	12 (27.27%)	<0.001

**Table 3:** Comparison of laboratory serum biomarkers and echocardiographic parameters for non-LAAT and LAAT groups in the entire cohort, and patients with CHA2DS2-VASc score ≤ 1 (female excluded) and patients without OAC, respectively.

Variables	Entire Cohort			Patients without OAC			Patients with CHA2DS2-VASc Score ≤ 1		
	Non-LAAT (*n* = 493)	LAAT (*n* = 50)	*p*-Value	Non-LAAT (*n* = 331)	LAAT (*n* = 44)	*p*-Value	Non-LAAT (*n* = 228)	LAAT (*n* = 20)	*p*-Value
**NT-proBNP (ng/L)**	649.90 (231.00–1310.70)	1352.90 (508.50–2828.00)	<0.001	648.90 (196.50–1261.80)	1404.00 (583.50–2996.30)	<0.001	501.30 (178.80–1021.50)	764.40 (273.70–2724.20)	0.196
**cTnI (ng/L)**	8.00 (1.00–10.00)	10.00 (6.00–22.00)	<0.001	8.00 (1.00–10.00)	11.00 (6.00–25.00)	<0.001	1.00 (1.00–10.00)	7.00 (3.00–21.00)	0.027
**eGFR (mL/min/1.73 m^2^)**	87.97 ± 22.81	79.89 ± 21.52	0.017	88.86± 23.30	81.68 ± 21.95	0.054	92.00 ± 21.67	83.28 ± 21.74	0.085
**Uric Acid (mmol/L)**	342.00 (281.50–414.00)	370.00 (314.50–480.00)	0.065	346.30 (284.60–412.55)	370.50 (318.75–480.00)	0.070	346.65 (274.25–401.25)	414.50 (335.50–483.50)	0.005
**TC (mmol/L)**	4.11 ± 0.95	4.04 ± 1.17	0.606	4.21 ± 0.96	4.08 ± 1.18	0.437	4.35 ± 0.84	4.65 ± 1.33	0.321
**LDL-L (mmol/L)**	2.04 ± 0.63	2.24 ± 0.75	0.038	2.10 ± 0.62	2.28 ± 0.73	0.082	2.18 ± 0.54	2.78 ± 0.62	<0.001
**TG (mmol/L)**	1.20 (0.88–1.63)	1.29 (0.99–1.67)	0.480	1.26 (0.91–1.69)	1.23 (0.98–1.67)	0.955	1.32 (0.96–1.73)	1.35 (1.06–1.89)	0.726
**LAD (mm)**	38.94 ± 5.38	42.14 ± 6.67	<0.001	38.55 ± 5.56	42.32 ± 6.90	<0.001	37.71 ± 5.38	39.70 ± 5.54	0.124
**LVEF (%)**	65.29 ± 9.47	59.92 ± 11.91	0.003	65.58 ± 9.85	59.93 ± 12.66	<0.001	65.02 ± 8.82	59.50 ± 10.64	0.009

**Table 4:** Characteristics of patients who suffered thrombotic events or death during follow-up.

ID	Sex	Age (Years)	Anticoagulants	CHA2DS2-VASc Score	Previous Thrombotic Events	Review of TEE	Outcomes	Time to Event (Months)
1	Female	67	Rivaroxaban 15 mg qd	6	Yes	No	Arterial embolism of lower extremities	13
2	Male	70	Warfarin 2.5 mg qd	6	Yes	No	Stroke	58
3	Female	81	Warfarin 1.25 mg qd	4	No	No	Death for unknown reason	12
4	Male	32	Warfarin 2.5 mg qd	1	No	No	Death due to end-stage heart failure	23

## Data Availability

The data are available upon request by contact with the corresponding author.
